# Phytochemical Profile and Bioactive Potential of *Hampea rovirosae Standl*.: Antioxidant, Antimicrobial, and Carbohydrate-Hydrolyzing Enzyme Inhibitory Activities

**DOI:** 10.3390/cimb48030327

**Published:** 2026-03-19

**Authors:** Maria Candelaria Tejero-Rivas, José Rodolfo Velázquez-Martínez, Minerva Aurora Hernández-Gallegos, Angelica Alejandra Ochoa-Flores, Rodolfo Osorio-Osorio, Juan Guzmán-Ceferino, Emmanuel Cabañas-García, Josafat Alberto Hernandez-Becerra

**Affiliations:** 1 Academic Division of Agricultural Sciences, Juárez Autonomous University of Tabasco, Carretera, Villahermosa-Teapa Km 25, Villahermosa CP 86298, Mexico; maria.tr@villlahermosa.tecnm.mx (M.C.T.-R.); angelica.ochoa@ujat.mx (A.A.O.-F.); rodolfo.osorioo@gmail.com (R.O.-O.); juan.guzman@ujat.mx (J.G.-C.); 2 National Technological Institute of Mexico, Villahermosa Campus, Villahermosa-Frontera Highway, Km. 3.5 Industrial City of Villahermosa, Villahermosa CP 86010, Mexico; 3 Multidisciplinary Academic Division of Jalpa de Méndez, Juárez Autonomous University of Tabasco, State Highway Villahermosa-Comalcalco Km. 27+000 s/n Ranchería Ribera Alta, Jalpa de Méndez CP 86205, Mexico; 4 Unidad Professional Interdisciplinaria de Ingeniería Campus, Zacatecas-Instituto Politécnico Nacional, Calle Circuito del Gato No. 202, Col. Ciudad Administrativa, Zacatecas CP 98160, Mexico; ecabanasg@ipn.mx; 5 Food Technology Division, Tabasco University of Technology, Carretera, Villahermosa-Teapa Km. 14.6, Parrilla II, Tab, Villahermosa CP 86288, Mexico; jahernandez.tc@uttab.edu.mx

**Keywords:** extracts, hyperglycemia, phytochemicals, metabolites, malvaceae, bioactives

## Abstract

*Hampea rovirosae Standl*. is traditionally used by local communities to treat infections, pain-related conditions, and to reduce blood sugar levels. In this investigation, we produced aqueous, ethanolic, and hydroethanolic extracts of *H. rovirosae* and assessed their antioxidant, antibacterial, and antihyperglycemic properties in addition to their phytochemical profiles and contents. The phytochemical characterization was performed through a targeted chromatographic and mass spectrometric analysis of phenolic compounds and the quantitation of total phenolic content (TPC), total flavonoid content (TFC), and total tannin content (TTC) by spectrometric assays. The antioxidant capacity was assessed using the DPPH, ABTS, and FRAP assays, and the antibacterial activity was determined by disk diffusion (DD) and minimum inhibitory concentration (MIC) methods. In addition, antihyperglycemic activity was evaluated by inhibiting α-amylase and α-glucosidase. Phytochemical analysis was performed using high-performance liquid chromatography coupled with mass spectrometry (HPLC-MS), employing a targeted analysis approach based on comparing retention times and fragmentation patterns with standards and databases. This analysis revealed a phytochemical profile dominated by phenolic compounds, with quercetin-3-glucoside (155,930.2), caffeic acid (134,399.1), catechin (98,408.8), procyanidin B2 (85,661.7), protocatechuic acid (83,824.3), and epicatechin (53,704.1) being the major metabolites. The hydroethanolic extract exhibited the highest phenolic (426.70 mg GAE/g), flavonoids (119.17 mg CE/g), and tannin (324.46 mg GAE/g) contents, as well as the strongest antioxidant capacity in the DPPH and FRAP assays. Regarding the antibacterial effects, the aqueous extract inhibited *Salmonella typhimurium* and *Escherichia coli*, while the hydroethanolic extract was active against *S. aureus, B. cereus,* and *B. subtilis*. In enzyme inhibition assays, the hydroethanolic extract showed strong α-glucosidase inhibition and moderate α-amylase inhibition. The findings provide preliminary scientific evidence of the antioxidant and biological activities of *Hampea rovirosae* in vitro, supporting its traditional use, which should be validated through vivo trials.

## 1. Introduction

Humans have depended on plants since ancient times, not only as sources of food and shelter but also as essential resources for treating diseases and traditional knowledge has been passed down from generation to generation [1,2]. Due to their content of bioactive substances and potential for drug development and discovery, medicinal plants are currently of significant scientific interest. In this regard, an estimated 25% of contemporary medications are derived directly or indirectly from plants [3,4].

Several botanical families are widely recognized for their medicinal value, including Lamiaceae, Asteraceae, Fabaceae, Rutaceae, Zingiberaceae, Rosaceae, Apiaceae, and Malvaceae [5,6,7]. These families are sources of bioactive compounds that provide therapeutic effects such as analgesic, anti-inflammatory, antihypertensive, antidiabetic, anticancer, antibacterial, and antioxidant activities [8,9]. In particular, species belonging to the Malvaceae family have traditionally been used to treat various ailments, including intestinal disorders, bacterial infections and inflammatory processes [10,11]. In addition, several members of this family have been reported to exhibit antioxidant and hypoglycemic activities, which have been associated with the presence of certain of phenolic bioactive molecules and their ability to inhibit intestinal glucose absorption [12,13,14].

Among the most diverse and most studied metabolites are phenolic molecules, which encompass multiple structural sub-classes [15]. These compounds are well known for their antioxidant capacity, as they can neutralize free radicals responsible for oxidative stress and the emergence of degenerative diseases such as cancer, diabetes, and cardiovascular disorders [16,17]. In addition to their antioxidant properties, phenolic compounds have demonstrated antibacterial activity and offer advantages, such as structural diversity and the possibility of functional modification, which can increase their biological effectiveness [18].

Bacterial contamination occurs when pathogenic microorganisms proliferate in different matrices, such as food, pharmaceuticals, cosmetics, or clinical environments. This problem is often associated with factors such as the use of contaminated water, improper handling, or poor aseptic conditions during production or processing [19,20,21]. To address this problem, various phytochemicals have been shown to have significant antibacterial effects by inhibiting the growth and proliferation of pathogenic microorganisms [22]. This is particularly relevant considering that, at present, bacterial resistance to antibiotics has become a global threat to public health [23,24]. In this context, the search for new antibacterial agents, particularly those of natural origin, has become very important. These compounds have a wide structural diversity and the ability to act through multiple mechanisms of action which can reduce the likelihood of developing bacterial resistance [25,26].

Similarly, interest in bioactive compounds of plant origin has also increased in the context of chronic metabolic diseases, such as diabetes mellitus (DM). This disease is one of the world´s health problems and is characterized by the inability of individuals to regulate their blood glucose levels [27,28]. According to the International Diabetes Federation (IDF), more than 400 million adults currently live with this chronic disease, and this number is projected to increase in the coming years [29]. Although there are various pharmacological treatments available to control the disease, interest in natural therapeutic alternatives has increase due to their accessibility, cultural acceptance, and lower incidence of adverse side effects [30].

Within the Malvaceae family, *Hampea rovirosae Standl*., commonly known as “Majahua”, is a tree species that grows wild in tall and medium evergreen and semi-evergreen forests in southeastern México [31]. It is characterized by broad, dark green leaves, white flowers, and grayish-green capsule fruits; it is also commonly grown in gardens and domestic courtyards [32]. Traditionally, this plant is used for medicinal purposes in various Mexican communities. Its leaves are the most-used part and, according to local tradition, are used in cold preparations for the treatment of wounds, headaches, gastritis, and mumps [33]. In addition, according to traditional knowledge, these preparations are also to help reduce blood glucose levels.

Despite its ethnomedical relevance, the scientific information available on *Hampea rovirosae* is limited. Although several species of the Malvaceae family have been studied for their phytochemical composition and biological activities, there are currently no studies that comprehensively describe the phenolic profile of *H. rovirose* or systematically evaluate its antioxidant, antibacterial, and antihyperglycemic activities. Consequently, the bioactive potential of this species remains largely unexplored. Therefore, the bioactive potential of this species remains largely unexplored. Based on its traditional use and the biological properties reported for other members of the Malvaceae family, it is hypothesized that extracts obtained from *Hampea rovirosae* leaves contain bioactive phenolic compounds whose concentration depends on the extraction method, and that these compounds contribute significantly to their antioxidant, antibacterial and antihyperglycemic activities.

Therefore, the objective of this study was to determine the content of total phenolic compounds, flavonoids, and tannins in aqueous (AE), ethanolic (EE), and hydroalcoholic (HEE) extracts of *Hampea rovirosae Standl*. leaves, as well as to evaluate their antioxidant, antibacterial, and antihyperglycemic activities. The results of this study may contribute to the scientific validation of the traditional use of this species and provide relevant information for the identification of bioactive compounds with potential application in the development of new therapeutic strategies against chronic diseases and bacterial infections.

## 2. Materials and Methods

### 2.1. Chemicals

All reagents employed were of analytical grade. Chemicals of standard reagents used for phytochemical and antioxidant assays, including Folin–Ciocalteu, catechin, gallic acid, PVPP (polyvinylpolypyrrolidone), DPPH• (2,2-diphenyl-1-picrylhydrazyl), ABTS•+(2,2′-azino-bis(3-ethylbenzothiazoline-6-sulfonic acid)), Trolox (6-hydroxy-2,5,7,8-tetramethylchroman-2-carboxylic acid), potassium persulfate, and TPTZ (2,4,6-tripyridyl-S-triazine) were purchased from Sigma-Aldrich^®^ (St. Louis, MO, USA). In addition, for enzymatic activity, porcine pancreatic α-amylase, tris-HCl, soluble starch, and DNS (3,5-dinitrosalicylic acid) were also purchased from Sigma-Aldrich^®^ (St. Louis, MO, USA). Na_2_CO_3_, NaNO_2_, NaOH, HCl, sodium acetate, AlCl_3_, acetic acid, DMSO (dimethyl sulfoxide), and FeCl_3_ were obtained from Meyer^®^ (Mexico, Mexico). Brain–heart infusion broth (BHI) was obtained from BD Dixon™ (Mexico, Mexico).

### 2.2. Pathogenic Bacteria

Antibacterial assays were performed using reference strains of *Salmonella typhimurium* (ATCC 14028)*, Escherichia coli* (ATCC 25922), *Bacillus cereus* (ATCC 11778), *Bacillus subtilis* (ATCC 6639) and *Staphylococcus aureus* (ATCC 25923). These strains were kindly provided by the Department of Food and Biotechnology, Faculty of Chemistry, National Autonomous University of Mexico.

### 2.3. Collection of Plant Material

The leaves of *Hampea rovirosae Standl.* were collected in April 2023 in the community of Ixtanpagajoya, Chiapas, Mexico (17°28′00″ N 92°59′00″ W). The collection was carried out at a single site during the flowering period, with the aim of ensuring adequate taxonomic identification of the species. To obtain the extracts, approximately 20 kg of mature, fully expanded leaves in good condition were collected. Botanical identification and taxonomic authentication were conducted at the Herbarium of the Academic Division of Biological Sciences, Juárez Autonomous University of Tabasco (folio No. 037178). After collection, the plant material was rinsed to remove surface impurities and residual moisture was eliminated using a manual centrifugation process. Drying was carried out at ambient temperature (25 °C) for five days in a controlled environment equipped with a window-type dehumidifier (LG^®^ Electronics, Seoul, South Korea, MODEL D451WH SE1). Subsequently, the dried leaves were milled, passed through a No. 60 mesh sieve, and the resulting powder was kept at 4 °C under light-protected conditions until analysis.

### 2.4. Targeted LC-MS Analysis of Flavonoids and Phenolic Acids in Hampea rovirosae Plant Material

Samples were processed as previously described in [34], with modifications to the liquid chromatography (LC) separation. Briefly, 50 mg of material was extracted using a 1:1 acetonitrile:methanol solution containing an internal standard. Samples were disrupted with a TissueLyserII (Qiagen, Germantown, MD, USA), centrifuged, and the resulting supernatants were transferred to new tubes and dried using a SpeedVac vacuum concentrator (TermoFisher Scientific, Carlsbad, CA, USA). Pellets were reconstituted in 15% methanol and analyzed by LC–MS using a Shimadzu Nexera 2 LC system coupled to a Sciex QTRAP 6500+ mass spectrometer (Sciex, Framingham, MA, USA) equipped with a TurboIonSpray (TIS) electrospray ion source. Flavonoids were separated on an ACCQ-TAG Ultra C18 column (1.7 µm, 2.1 × 100 mm; Waters) at 0.4 mL/min using a gradient of mobile phase A (0.1% formic acid in water) and mobile phase B (0.1% formic acid in acetonitrile). Data were acquired in both positive and negative ion modes using Analyst software (version 1.7.2). Metabolites were quantified using MRM transitions previously optimized with authentic standards [35]. External calibration curves were generated using serially diluted standards containing fixed concentrations of the internal standard. Supplementary Table S1 shows the LOD (limits of detection), ULOQ, and LLOQ (upper and lower limits of quantification, respectively) of the target compounds. Supplementary Figure S1 presents the total ion chromatogram of *H. roviroseae*.

### 2.5. Extraction Procedure for Hampea rovirosae Leaves Using Different Solvents

The extracts were obtained from the powdered *H. rovirosae* leaves using three different extraction solvents: deionized water (aqueous extract, AE), ethanol (ethanolic extract, EE), and ethanol:deionized water (70:30 *v*/*v*, hydroethanolic extract, HEE). For AE, the powder was mixed with deionized water and heated in a water bath at 90 °C for 30 min. The mixture was filtered through a Whatman No. 1 filter paper, followed by microfiltration using a nitrocellulose membrane (0.45 µm pore size). The water from the filtered extract was removed by lyophilization (Scientz 10N Freeze Dryer^®^ Ningbo Scientz Biotechnology Co., Ltd., Ningbo, China). To obtain the EE and HEE extracts (2%, *w*/*v*), the powdered material was macerated with the corresponding solvent at a solid-to-solvent ratio of 1:50 (*w*/*v*) under constant (48 h, 150 rpm). Each mixture was filtered through Whatman No. 1 filter paper and centrifuged at 8000× *g* (Hermle Z 323K, Hermle Labortechnik GmbH, Wehingen, Germany). The ethanol was removed using a rotary evaporator at 55 °C, while the residual water from the HEE was eliminated in a vacuum oven (Felisa FE-291^®^ oven, Felisa, Mexico, Mexico) at 60 °C. The resulting dry extracts were stored at 4 °C in amber bottles until use.

### 2.6. Total Phenolic Content (TPC)

Total phenolic content (TPC) was determined following a modified Folin–Ciocalteu procedure based on Singleton et al. [36] Briefly, the extract (0.125 mL) was reacted with diluted Folin–Ciocalteu reagent (1:10 *v*/*v*), and sodium carbonate solution (7.5%). After incubation for 45 min at ambient temperature under light-protected conditions, absorbance was recorded at 760 nm using UV-Vis spectrophotometer (UV-1800 Rayleigh^®^). Quantification was achieved using with gallic acid calibration curve (10 to 100 µg/mL) and results were expressed as mg gallic acid equivalents per g of dry extract (mg GAE/g dry extract). All analyses were conducted in triplicate.

### 2.7. Total Flavonoid Content (TFC)

Total flavonoid content (TFC) was quantified using a colorimetric assay adapted from Barros et al. [37]. Briefly, aliquots of the extract (0.2 mL) were combined with distilled water and sodium nitrite solution (5%), followed by the addition of aluminum chloride (10%). After sequential incubations periods, the reaction was alkalinized with sodium hydroxide (10%), and absorbance was recorded at 510 nm using a UV-Vis spectrophotometer. Quantification was performed using a catechin calibration curve (100 to 500 µg/mL) and results were expressed as mg of (±) catechin equivalents (CE) per g of dry extract (mg). All determinations were carried out in triplicate.

### 2.8. Total Tannin Content (TTC)

Total tannin content was estimated using the method of Makkar, [38] with slight modifications. 100 µL of each extract, 100 mg of polyvinylpolypyrrolidone and distilled water were combined, swirled, and then incubated for 15 min at 4 °C. After stirring, the liquid was centrifuged for 10 min at 3000 rpm. After centrifugation, the supernatant, containing non-tannin phenolic was recovered, and phenolic content was determined, as previously described. Tannin level were calculated by difference and expressed as mg gallic acid equivalents per g of dry extract (mg GAE/g dry extract).

### 2.9. Antioxidant Activity Determination

#### 2.9.1. DPPH• Radical Scavenging Activity

Antioxidant capacity was evaluated through a DPPH• radical scavenging assay adapted from Brand-Williams et al. [39]. Extract aliquots (200 μL) were reacted with a methanolic DPPH• solution (125 μM 80% methanol) and maintained under light-protected conditions at room temperature for 30 min. The decrease in absorbance was subsequently recorded at 517 nm. Inhibitory concentration (IC_50_) values, defined as the extract concentration required to neutralize 50% of the radicals, were calculated by regression and expressed in mg/mL. Radical scavenging activity was calculated according to the equation:

(1) % inhibition = (control Abs − sample Abs)/(control Abs) × 100

where

• Control Abs is the absorbance of the reagent mixture without adding sample or standard.
• Sample Abs is the absorbance of the mixture added with the sample or standard reagent.

#### 2.9.2. ABTS•+ Assay

Radical scavenging capacity was assessed using an ABTS•+ decolorization assay, adapted from Re et al. [40]. The ABTS•+ working solution was generated by reacting ABTS (7 Mm) with potassium persulfate (2.45 mM) in equal volumes and allowing the mixture to stabilize for 12–16 h under dark conditions at room temperature. Prior to analysis, the solution was diluted with methanol to obtain absorbance of 0.800 ± 0.05 at 734 nm. For the assay, extract aliquots (10 μL) were added to the ABTS•+ solution (990 μL), and absorbance was recorded at 734 nm after six minutes of incubation. Antioxidant activity was expressed as IC_50_ (mg/mL), calculated as described for the DPPH• assay (Equation (1)).

#### 2.9.3. Ferric Reducing Antioxidant Power (FRAP)

With minor adjustments, the Benzie & Strain [41] technique was used for the FRAP test. The FRAP reagent was freshly prepared by mixing acetate buffer (300 mM in pH 3.6), TPTZ solution (10 mM, in 40 mM HCl) and ferric chloride (20 mM) in a 10:1:1 (*v*/*v*/*v*) proportion. Extract (100 μL) were reacted with the FRAP reagent (3 mL), and absorbance was measured at 593 nm after a 30 min incubation. Quantification was performed using a Trolox calibration curve (200 to 1400 µmol), and results were expressed as µM per g of dry extract (µM TE/g dry extract).

### 2.10. Antibacterial Activity

#### 2.10.1. Disc Diffusion Assay

The antibacterial potential of the extracts was assessed by a disc diffusion (DD) assay following standard procedures [42]. The activity was tested against *Salmonella typhimurium*, *Staphylococcus aureus*, *Escherichia coli*, *Bacillus subtilis*, and *Bacillus cereus*. Bacterial strains were recovered from glycerol stock (−20 °C) and culture in brain–heart infusion (BHI) broth prior to analysis. Standardized inocula (10^8^ CFU/mL) were spread on nutrient agar plates, and sterile paper discs impregnated with 10 mg of each extract were place on the source. Disc containing amikacin (30 μg) were used as positive controls, while solvent-loaded disc served as negative controls [43]. Plates were incubated at 37 °C for 12–24 h after which antibacterial activity was determined by measuring inhibition zones diameter (mm).

#### 2.10.2. Minimum Inhibitory Concentration (MIC) and Minimum Bactericidal Concentration (MBC)

The microplate dilution method was used to find the MIC. For example, Mueller–Hinton (MH) broth was used to activate the bacterial strains *S. aureus*, *S. typhimurium*, *E. coli*, *B. subtilis*, and *B. cereus.* The experiment was conducted using sterile 96-well microplates (500 μL capacity). The extracts were applied starting at 60, 200, and 400 mg/mL at a serial dilution rate (1:1). Aqueous extracts were diluted with sterile distilled water, while ethanolic and hydroethanolic extracts were diluted with 10% sterile DMSO. In each well, 100 μL of inoculated MH broth and 100 μL of diluted extract or control (positive or negative) were added. As positive controls, amikacin and chloramphenicol were used at dilution rates of 2 to 0.015 and 4 to 0.03 mg/mL, respectively [44]. Sterile distilled water and 10% DMSO (10 to 0.0075%) make up the negative control. In the vortex shaker (IKATM), microplates were shaken for five minutes at 150 rpm. The microplates were incubated at 37 °C. for twenty-four hours. A Petri dish containing MH agar was inoculated with 10 μL of the well holding the MIC, and the mixture was incubated for 24 h. The MBC was determined from the non-turbid wells obtained in the MIC assay. Ten μL of each clear well was subcultured onto antimicrobial-free MH agar plates and incubated at 37 for 24 h. The MBC was defined as the minimum concentration at which 99.9% of the final inoculum was eliminated [45].

### 2.11. Antihyperglycemic Activity

#### 2.11.1. Inhibition of α-Amylase Activity

Utilizing the approach outlined by Ji et al. [46], the inhibition of α-amylase activity was ascertained. To do this, Tris-HCl buffer (0.5 M, pH 6.9) was combined with 0.2 mL of CaCl_2_ (0.01 M) and starch (2 mg) to create a substrate solution. After distributing 200 μL of the substrate solution into test tubes, were preincubated for five min at 37 °C. 100 μL of buffer solution, 200 μL of pig pancreatic α-amylase (2 U/mL), 200 μL of the extracts at different concentrations, 200 μL of dimethyl sulfoxide (DMSO) 50%, and dinitrosalicylic acid (500 μL) of 3.5–0.1% were then added. After 10 min of the reaction at 100 °C, the resultant mixture was allowed to stand for 15 min at room temperature. Acarbose was used as a positive control to measure the absorbances of the mixes at a wavelength of 540 nm. Equation (2) was used to calculate the inhibitory activity.

(2) % inhibition= [(Ac^+^) − (Ac^−^) − (As − Ab)]/[(Ac^+^) − (Ac^−^)] × 100

whereas Ac^−^ represents the absorbance when the enzyme does not react (solvent with enzyme exclusively), Ac^+^ represents the absorbance when the enzyme responds unhindered. Ab is the absorbance of a blank, and As is the absorbance when the enzyme reacts with the sample present.

#### 2.11.2. Inhibition of α-Glucosidase

The test was performed according to the methodology of Pistia-Brueggeman and Hollingsworth [47], with some modifications. Reaction mixtures comprising 350 μL of phosphate buffer (0.1 M, pH 6.8), 150 μL of the extract at several concentrations, and 30 μL of α-glucosidase (Saccharomyces cerevisiae) at 1 U/mL was preincubated at 37 °C for 20 min. 60 μL of a 10 mM pNPG (4-nitrophenyl-β-D-glucopyranoside) solution (substrate) was added to initiate the reaction, and the mixture was incubated for an additional 30 min. The reaction was terminated with sodium carbonate solution (0.05 M) and absorbance was recorded 405 nm using a spectrophotometer. For this test, acarbose served as a positive control. For every concentration, the trials were conducted three times. The IC_50_ values for the extracts and the positive control were determined, and the inhibitory activity of α-glucosidase was calculated using the same equation as for α-amylase inhibition.

### 2.12. Statistical Analysis

Date was analyzed using a completely randomized design. Results are expressed as mean ± standard deviation, with all assays conducted in triplicate. Statistical differences among treatments were evaluated by one-way analysis of variance (ANOVA), followed by Tukey’s post hoc test at a significance level of *p* < 0.05. Associations between antioxidant activity and the total phenolic content were examined using Pearson’s correlation coefficients (*p* < 0.05). IC50 values were estimated by linear regression analysis. All statistical procedures were performed using Statgraphics Centurion XIX version 19.1.2 software.

## 3. Results and Discussion

### 3.1. Liquid Chromatography–Mass Spectrometry Analysis of Hampea Rovirosae

Targeted LC-MS profiling revealed a diverse phenolic composition in *H. rovirosae*, allowing the detection and quantification of several phenolic constituents (Table 1). The most abundant compound was quercetin-3-glucoside (155,930.2 ng/g), followed by caffeic acid (134,399.1 ng/g), catechin (98,408.8 ng/g), and procyanidin B2 (85,661.7 ng/g). In addition, protocatechuic acid (83,824.3 ng/g) and epicatechin (53,704.1 ng/g) were also found.

Other identified metabolites included ferulic, gallic, chlorogenic, p-coumaric, syringic, vanillic, and cinnamic acids, as well as flavonoids including quercetin-3-glucoside, luteolin, apigenin, quercetin, quercetin-3-galactoside, kaempferol, and naringenin chalcone (see Table 1). Isoflavonoids such as genistein and daidzein were detected at low concentrations of 10.2 and 3.51 ng/g, respectively. Under the proposed analytical conditions of this study, naringenin, phloretin, resveratrol, and rutin were not detected.

Targeted UHPLC-MS analysis revealed that *H. rovirosae* has a chemical profile dominated by flavonoids and phenolic acids, which is closely correlated with various biological activities. High concentrations of quercetin-3-O-glucoside, together with caffeic, protocatechuic, and gallic acids, suggest that these metabolites may be responsible for the antioxidant and bioactive capacity of this plant species. This chemical profile is consistent with previous research reported in other species of the Malvaceae family. Such is the case which the presence of caffeic and protocatechuic acids, as well as quercetin flavonoids reported in the species *Hibiscus mutabilis* and *Malvaviscus arboreus* [48]. Similarly, in the species *Hibiscus roseus*, phenolic acids, ferulic and chlorogenic acids, as well as glycosylated flavonoids, including quercetin-3-O-glucoside, were reported [49]. It has been widely reported that these types of compounds exert various biological activities, including antioxidant, hepatoprotective, anti-inflammatory, antibacterial and antidiabetic effects, which are closely linked to their structural characteristics and redox-related interactions [50,51].

### 3.2. Extraction Yield

In the extraction of secondary metabolites, the choice of solvent is of vital importance, as it affects not only toxicity but also the efficiency of their recovery [52]. Table 2 shows the yield percentages of the extracts of *H. rovirosae* Standl. As can be seen, AE and HEE showed the highest yields (33.5 and 32.13%, respectively), which were significantly higher (*p* < 0.05) than EE (17.93%). The results suggest that mixing water into the extraction solvent facilitates the recovery of a greater amount of compounds. The lower efficiency observed with ethanol can be attributed to its limited affinity for some polar compounds, while mixed solvents, such as hydroethanol, provide a balance of polarity, and thus optimize the extraction process of compounds with different polarities.

### 3.3. Phytochemical Content of the Extracts

#### Total Phenolic Content (TPC), Total Flavonoid Content (TFC), and Total Tannin Content (TTC)

Phenolic compounds are secondary metabolites present in various parts of plants, and are recognized for their multiple health benefits [53]. Table 2 shows TPC, TFC, and TTC of *H. rovirosae* leaves. As indicated, the TPC was significantly higher (*p* < 0.05) in the hydroalcoholic extract (426.70 ± 1.06 mg GAE/g of dry extract), followed by the aqueous (252.15 ± 3.37 and 191.24 ± 3.71 mg GAE/g dry extract respectively).

Although no specific reports were found on *H. rovirosae*, evidence is available in the literature on other species of same family. In this context, Shadid et al. [14] reported TPC = 191.54 ± 2.84 mg GAE/g for methanolic extracts and TPC = 95.5 ± 2.67 mg GAE/g for aqueous of *M. oxyloba*. Similarly, Ezeako et al. [54] reported 91.64 ± 7.61 mg GAE/g in ethanolic extracts of *Sida linifolia* Linn (Malvaceae). These values are lower than those obtained in the present investigation, highlighting the influence of the type solvent on the content of phenolic compounds in plants [55]. Although water can serve as an effective polar solvent for extraction of bioactive compounds, the incorporation of alcohols, such as ethanol, significantly improves extraction efficiency by enhancing the solubilization of compounds of varying polarity [56,57].

The high phenolic content observed in this study could be linked to the possible antioxidant activity attributed to this species, since according to previous studies these compounds are good free radicals neutralizers [58]. In this context, several studies have identified phenolic compounds, such as ferulic and ellagic acids in some Malvaceae species, which are associated with different biological activities, such as antidiabetic, anti-inflammatory, antimicrobial, and antiallergic, among others [54,59]. Furthermore, comparisons with other species in the same family suggest that *H. rovirosae* has considerable potential for biological activities, reinforcing the need for further research to evaluate its relationship with specific biological properties [60].

Regarding the total flavonoid content analysis (Table 2), the HEE exhibited the highest value (119.17 ± 0.90 mg CE/g of dry extract; *p* < 0.05), followed by the aqueous (46.76 ± 1.32 mg CE/g dry extract) and ethanolic (53.53 ± 1.32 mg CE/g dry extract) extracts. The TFC in *H. rovirosae* HEE is comparable to that reported for *T. cordifolia* HEE, another Malvaceae species, which contained 139.30 ± 0.99 mg CE/g [61]. In contrast, Hamrita et al. [62] documented lower TFC values in *H. sabdariffa*, with 22.49 ± 1.04 and 16.3 ± 1.85 mg CE/g of dry extract in the aqueous and methanolic extracts, respectively. It should be noted that the HEE from *Hampea rovirosae* showed the highest TPC and TFC, highlighting the effectiveness of mixed solvents in obtaining higher bioactive compounds.

These compounds are flavonoids such as isoquercitrin and quercetin-3-O-beta-glucopyranosyl-glucoside, which have been identified as inhibitors of α-glucosidase, an enzyme involved in postprandial glucose regulation. In addition, protocatechuic acid, another relevant phytochemical, has been reported to exhibit antibacterial properties [13,63]. These findings support the antioxidant and biological activities attributed to flavonoids in species of the Malvaceae family, and suggest that *H. rovirosae* warrants further investigation for potential nutraceutical or pharmacological applications [64].

With regard the total tannin content (TTC), significant differences (*p* < 0.005) were observed between the HEE (Table 2), which had the highest concentration (324.46 ± 5.31 mg GAE/g dry extract), and the AE (196.39 ± 3.17 mgGAE/g dry extract) and the EE (142.83 ± 2.34 mg GAE/g dry extract). When comparing these values with those reported on other trials, the tannin content obtained in this study exceeds that reported for the methanolic extract of *Byttneria pilosa* [60], another member of the Malvaceae family, as well as the hydroethanolic extracts of *Cola nitida* reported by Sarr et al. in [65]. These results highlight the potential of the hydroalcoholic extract of *H. rovirosae* as a rich source of tannins, which can also be associated with relevant bioactive effects, such as antioxidant and antimicrobial activities, among others [66]. In this context, some condensed tannins, such as proanthocyanidins, have been relevant in the development of multiple biological activities [12].

Based on the phytochemical content results, it can be inferred that the polarity of the extraction solvent strongly influences the amount of TPC, TFC and TTC in the different extracts [67,68,69]. In this sense, hydroalcoholic extracts are widely recognized for their capacity to solubilize phenolic compounds with different polarities, thereby covering a broader polarity range than pure solvents. This property enables a more efficient and representative recovery of bioactive metabolites present in the plant material [59,61]. Similarly, it is important to note that differences in phenolic content could also be attributed to factors such as the intrinsic characteristics of each species, the extraction method, and processing conditions, all of which directly influence compound recovery [70,71,72]. Finally, targeted analysis using HPLC-MS allowed for the identification and quantification of a diverse set of phenolic compounds, including glycosylated flavonoids, flavan-3-ols, procyanidins, and phenolic acids, which constitute the major fraction of the extract’s phytochemical profile. These results are consistent with the high values obtained in the determination of total phenol, flavonoid, and tannin content, suggesting that the metabolites identified make a significant contribution to these phytochemical fractions.

### 3.4. Antioxidant Activities

#### DPPH•, ABTS•+, and FRAP Free Radical Scavenging Activity

Given that the antioxidant activity of plants is attributed to secondary metabolites that neutralize free radicals associated with disease development [17], this study evaluated the antioxidant activity of the different extracts using the DPPH, ABTS, and FRAP assays (Table 3). In the DPPH• assay, HEE had the lowest IC_50_ value (0.110 ± 0.00 mg/mL), compared to AE (0.196 ± 0.00 mg/mL) and EE (0.252 ± 0.01 mg/mL) extracts, indicating greater antioxidant capacity (*p* < 0.05). This behavior can be correlated with the higher TPC, TFC, and TTC values observed in HEE, highlighting the influence of the content of these metabolites and the choice of extraction solvent as key factors in radical scavenging activity via hydrogen or electron donation [73,74].

Complementarily, the authors have reported IC_50_ values using the DPPH assay in different species of the Malvaceae family, highlighting the variability associated with the solvent type and plant matrix. In this regard, Das et al. [12] presented an IC_50_ = 0.027 mg/mL for the ethanolic extract of *Ceiba pentandra*, while Silué et al. [75] reported values of 0.184 mg/mL for its hydroethanolic extract. In another study, Nwankwo et al. [76] documented IC_50_ = 0.64 mg/mL for *Sida linifolia* aqueous extract and 0.51 mg/mL for ethanolic extract. Similarly, Rainatou et al. [77] reported an IC_50_ = 0.106 mg/mL for the methanolic extract of *Hibiscus panduriformis*. These values allow us to compare the extracts evaluated in this study with those of other Malvaceae species, thus reinforcing the relevance of solvent selection for obtaining phytochemical compounds in this type of plant and, therefore, their high in the bioactive potential.

Regarding the ABTS•+ assay, EE showed the lowest IC_50_ (0.295 ± 0.00 mg/mL), indicating the greatest capacity to deactivate this radical, compared to HEE (0.507 ± 0.00 mg/mL) and AE (0.647 ± 0.01 mg/mL). These differences could be attributed to the varying affinities that solvents have for antioxidant compounds of different polarity, since ABTS•+ can be neutralized by both hydrophilic and lipophilic compounds [78].

Previous studies on ABTS•+ scavenging in Malvaceae species aid in contextualizing our findings. Rainatou et al. [77] reported IC_50_ = 3.721 mg/mL and 3.520 mg/mL in methanolic extracts of *W. rostrata* and *H. panduriformis*, respectively. Ezeako et al. [54] observed an IC_50_ = 0.75 mg/mL in ethanolic fractions *of Sida linifolia*, while El-Shiekh et al. [79] documented an IC_50_ = 0.71 mg/mL in methanolic extracts of *H schizopetalus*. Notably, Silué et al. [75] reported a low IC_50_ = 0.024 ± 0.00 mg/mL in hydroethanolic extracts of *Ceiba pentandra*, reinforcing the effectiveness of hydroethanol for extracting antioxidant compounds; nevertheless, it is also important to take into account the source of plant material and the intrinsic characteristics of each plant species.

Interestingly, in our study, the best activity in the DPPH and ABTS assays was observed with extracts prepared using different solvents, highlighting the broader antioxidant potential of *H. rovirosae*. This variation, in addition to being due to the presence of different phytochemical compounds extracted by each solvent, could also be attributed to the different ways in wich the mechanisms of action of both assays are carried out. In this sense, using DPPH, the mechanisms is mainly based on hydrogen atom transfer (HAT), while the ABTS method can proceed via both HAT and single electron transfer (SET), being also more sensitive to hydrophilic compounds [80,81]. Therefore, the combined evaluation of both assays provides a better understanding of the multiple pathways through which plant extracts neutralize free radicals.

As for the reducing power determined by the FRAP assays, unlike DPPH and ABTS, which express antioxidant activity as IC_50_ values (where lower values indicate greater radical scavenging capacity) the FRAP method directly measures ferric reducing antioxidant power and is expressed as Trolox equivalents. The measurement is based on the ability of these compounds to donate electrons and reduce the ferric ion complex (Fe^3+^), which generates a color change proportional to the concentration of antioxidants [48,62]. Therefore, the higher FRAP values indicate greater reducing power. In our study, the HEE extract showed the highest reducing capacity (2096.66 ± 80.82 µmol TE/g) (see Table 3), followed by the ethanolic and aqueous extracts (1032.66 ± 8.08 and 1026.66 ± 44.06 µmol TE/g, respectively; *p* < 0.05). This behavior, as with the previous methods may be related to the higher concentration of phenolic compounds quantified in the HEE, which act as reducing agents by donating electrons to the ferric ion (Fe^3+^) and, in addition, neutralize free radicals [17].

The results obtained with this method are higher than those presented in previous studies of other Malvaceae plants. Examples of this are those described by Silué et al. [75], who found a reducing capacity of 1122.55 µmol TE/g in hydroethanolic extracts of *C. pentandra*, underscoring the remarkable antioxidant capacity observed in the HEE of *H. rovirosae*. Similarly, Saleem et al. [82] reported values of 492.02 µmol TE/g in methanolic extracts of *A. figarianum*, while Ozturk et al. [83] described ferric reductions of 97.28 µmol TE/g and 112.71 µmol TE/g for methanolic and aqueous extracts of *A. fasciculiflora*, respectively. Collectively, these references reinforce the relevance of phenolic content and solvent type in determining the reducing capacity, allowing us to gauge the high efficacy of the hydroethanolic extract evaluated in this study.

In general, extracts of *Hampea rovirosae* Standl. exhibited a consistent antioxidant profile in all the tests performed, supporting their potential as a possible source of bioactive compounds in nutraceutical or pharmacological applications aimed at preventing oxidative. In this regard, this antioxidant profile may be related to the presence of caffeic, protocatechuic and chlorogenic acids, identified by HPLC–MS. These compounds are well known for their ability to reduce or eliminate reactive species, and act as reducing agents, which could collectively contribute to the antioxidant activity observed. On the other hand, the concordance between the HPLC–MS phytochemical profile, the TPC, TFC, TTC, and the antioxidant activity values obtained suggests that the effect exerted by the evaluated extracts is not attributable to a single compound, but rather to the combined and synergistic action of multiple phenolic metabolites [84,85].

### 3.5. Correlation Between TPC and Antioxidant Activity

To evaluate the influence of TPC of the aqueous, ethanolic, and hydroethanolic extracts of *H. rovirosae Standl* on antioxidant activities, it was necessary to calculate Pearson’s linear correlation coefficients (r^2^) for the three assays. A strongly negative correlation (*p* < 0.001, Table 4) was found between TPC and antioxidant activities and DPPH (r^2^ = −0.9887) and ABTS assays (r^2^ = −0.9683). This can explained because a higher content of phenolic compounds is associated with a lower IC_50_ value and, therefore, greater antioxidant activity, according to the mechanism of action, to neutralize free radicals by both methods evaluated [78,86].

For the FRAP assay, a positive and highly significant correlation was observed (r^2^ = 0.9869, *p* < 0.001). This result is consistent with the mechanism of action presented in this type on test, in which the extracts evaluated demonstrate their antioxidant capacity to reduce ferric ion (Fe^3+^) under acidic conditions [87]. It should be noted that the selection of the solvent remains important, as it has a considerable influence on the phenolic content and, therefore, on antioxidant potential. Both water and ethanol, as well as their mixtures, have proven to be efficient solvents for extracting phenolic compounds, especially in Malvaceae species [88].

Currently, the influence of phenolic content on antioxidant activity is a topic of great scientific and applied interest due to the ability of these compounds to reduce or neutralize free radicals and therefore oxidative stress [89,90]. Understanding and exploiting this relationship could optimize the use of these metabolites for the benefit of human health, taking into account that the antioxidant potential of these molecules depends largely on their structural feature and the specific neutralization mechanism involved [91,92].

### 3.6. Antibacterial Effect of Hampea Rovirosae Leaves Extracts

In Table 5, it can be observed that the aqueous extract exhibited inhibitory activity against *S. typhimurium* and *E. coli*, with inhibition zones of 9.1 ± 0.28 and 9.15 ± 0.49 mm, respectively, and MIC values of 200 mg/mL for both. On the other hand, none of the tested strains exhibited antibacterial activity when exposed to the ethanolic extract. The hydroethanolic extract was active only against *S. aureus* (9.95 ± 0.0 mm), *B. cereus* (12.15 ± 1.20 mm) and *B. subtilis* (14 ± 1.27 mm), reaching MIC of 60, 200, and 400 mg/mL, respectively. In all cases where activity was detected, the MBC values coincided with those of MIC, indicating a bactericidal effect and not just growth inhibition. The hydroethanolic extract showed bactericidal activity against the Gram-positive strains evaluated, while the AE showed a bactericidal effect against Gram-negative bacteria.

The antibacterial differences observed between AE and EHE could be attributed to the presence and content of the different types of secondary metabolites extracted by each of the solvents used [93], as well as the bacterial structure. In this regard, phenolic compounds are recognized molecules with bactericidal activity through several mechanisms of action. The most widely proposed include cell membrane alteration, interference in biofilm formation, enzyme activity inhibition, and impairment in DNA replication [18,94].

The difference in antibacterial effect observed between AE and HEE can be attributed, in part, to the way in which the bioactive compounds present interact with bacterial structures. In the case of Gram-negative bacteria, the presence of an outer membrane containing porin-type proteins restricts the passage of molecules, allowing only hydrophilic compounds to enter, which are mainly found in AE [95,96]. In contrast, in Gram-positive bacteria, the absence of this outer membrane facilitates the diffusion of both hydrophilic and hydrophobic compounds through the cell wall [97,98]. In this context, AE, due to their high polarity, are characterized by containing mainly hydrophilic phenolic compounds, such as phenolic acids and flavonoids. In contrast, hydroalcoholic extraction systems allow the recovery of metabolites with a wider range of polarity, including polyphenols and glycosides, which could explain the greater activity observed in these extracts against the microorganisms evaluated [99,100].

The results obtained through HPLC-MS-directed analysis allowed for the identification and quantification in *H. rovirosae* of a group of flavonoids, procyanidins, and phenolic acids that have been previously associated with antimicrobial activity [101,102]. Specifically, the targeted assay confirmed the presence of protocatechuic acid (83,824.3 ng/g), catechin (98,408.8 ng/g), epicatechin (53,704.1 ng/g), procyanidin B2 (85,661.7 ng/g), and flavonoids such as quercetin-3-glucoside (155,930.2 ng/g), compounds widely reported as modulators of bacterial cell membrane permeability [103,104]. Furthermore, the high abundance of procyanidins and phenolic acids, as confirmed by HPLC-MS analysis, suggests a possible synergistic effect between these metabolites, since they have been reported to form complexes with bacterial membrane proteins, altering their structural integrity and function [105,106].

The results obtained in this study can be compared with those from other Malvaceae species, which have also presented antibacterial activity. For instance, the aqueous and hydroethanolic extracts of *Sida acuta*, *Hibiscus sabdariffa*, and *Piliostigma senegalensis* have shown activity against *S. aureus*, *E. coli*, *S. typhimurium*, and *B. cereus* according to [107,108,109,110,111], which is consistent with our observations on *H. rovirosae*, suggesting that different members of the Malvaceae family share metabolites with antimicrobial activity, in which the effectiveness depends on the extraction solvent [93]. However, it is important to note that the MIC values obtained (200–400 mg/mL) in the present study were high, limiting their direct clinical relevance. These concentrations are characteristic of crude extracts, where active compounds are present in relatively low abundance. Therefore, the observed antibacterial activity should be interpreted as preliminary in vitro evidence. Further fractionation and isolation of the active components is required to better determine the therapeutic potential of this species. While the presence of secondary metabolites in *H. rovirosae* leaves is consistent with its ethnomedical use, additional pharmacological and toxicological evaluations are required before proposing its application in the treatment of wound infections.

### 3.7. Inhibition of digestive enzymes

#### 3.7.1. The Inhibitory Effect of Alpha-Amylase

During digestion, alpha-amylase plays a crucial role in converting complex carbs into simple sugars [112]. In this regard, there is constant interest in identifying natural resources that can inhibit this enzyme and contribute to the control of hyperglycemia. Table 6 displays the findings of *H. rovirosae* Standl in vitro enzymatic inhibitory activity.

The HEE, AE, and EE extracts showed inhibition percentages of 15.8%, 14.3%, and 9.9%, respectively (dose of 1 mg/mL). At the same dose, acarbose, which served as a positive control, demonstrated noticeably higher inhibition (78%, *p* < 0.05) concentration. The IC_50_ values obtained was 3.08, 4.65, and 3.06 mg/mL for AE, EE, and HEE, respectively, while that for acarbose was 0.37 mg/mL. When comparing the IC_50_ values obtained for the extracts of *H. rovirosae* Standl with those reported for other plant of the Malvaceae family, it can be seen that in the case of AE and HEE, their inhibitory activity against α-amylase is similar to that reported in extracts of *Melochia coromandelianum* and *Abelmoschus esculentus* (IC_50_ = 3.61 and 3.54 mg/mL, respectively). Meanwhile, when compared with species such as *Sida linifolia* (IC_50_ = 0.56 mg/mL) and *Abutilon indicum* (IC_50_ = 2.08 mg/mL), its inhibitory activity is clearly lower [54,113,114]. Although *H. rovirosae* Standl extracts exhibited inhibitory activity against α-amylase, the values obtained indicate moderate inhibition compared to acarbose. However, this behavior could be considered pharmacologically favorable since excessive inhibition of α-amylase is often associated with adverse gastrointestinal effects [115].

The inhibitory activity observed in *H. rovirosae* extracts could be closely related to their phytochemical profile, abundant in flavonoids and phenolic compounds, secondary metabolites widely associated with antihyperglycemic effects. In this context, the targeted analysis revealed the presence of phenolic acids at high concentrations, notably caffeic acid (134,399.1 ng/g), protocatechuic acid (83,824.3 ng/g), and chlorogenic acid (8486.1 ng/g), compounds that have demonstrated a high affinity for digestive enzymes involved in carbohydrate metabolism, acting individually or synergistically [116]. Furthermore, previous studies have demonstrated that chlorogenic acid, a common phenolic compound found in plants, can interact directly with the catalytic site of the α-amylase enzyme through hydrogen bonding, thus interfering with its enzymatic activity [117].

Similarly, ellagitannins such as chingiitannin A have been identified, which exhibit greater inhibitory activity than acarbose against the α-amylase enzyme by forming hydrogen bonds at the enzyme’s allosteric sites [118]. Regarding flavonoids, the *H. rovirosae* profile showed a high concentration of quercetin-3-glucoside (155,930.2 ng/g), as well as the presence of quercetin (942.4 ng/g) and kaempferol (408.9 ng/g), compounds previously reported in Malvaceae plants with inhibitory activity on the α-amylase enzyme [119]. Likewise, flavonols and flavones, such as myricetin, which are also present in this family, have been associated with regulating blood glucose levels [120]. Although the exact mechanism underlying the inhibitory effects these flavonoids have not been fully elucidated, experimental evidence and molecular docking models indicate that they may interact covalently with the enzyme active site by forming quinone-like structures, thereby competing with carbohydrates, decreasing their digestion [121,122].

#### 3.7.2. Alpha-Glucosidase Inhibitory Effect

Like α-amylase, α-glucosidase is important for the final absorption of glucose and is found on the brush border of enterocytes [123,124]. It has been demonstrated that simultaneously inhibiting both enzymes is a successful for lowering postprandial glucose levels [125].

Table 6 shows the inhibition of α-glucosidase by extracts of *H. rovirosae* Standl (dose of 50 µg/mL). HEE showed the highest inhibition value, with 94.73%, significantly higher than the drug acarbose at the same concentration (inhibition: 0.36%, *p* < 0.05). Following these values are the EE (90.12%) and AE (36.44%). Based on the above, it is important to note that HEE showed greater inhibitory efficacy against α-glucosidase at a lower concentration (50 µg/mL) than against α-amylase, demonstrating a more pronounced activity on the latter enzyme. This difference in inhibitory efficacy could be attributed to the phytochemical content of the extract, the mechanism of action, and the interaction of these with the different catalytic site [126,127].

The IC_50_ results obtained reinforce the inhibitory potential of HEE, as it presented the lowest value (15.74 µg/mL), followed by EE (25.76 µg/mL) and AE (66.01 µg/mL). All of the above were significantly lower than the value recorded for acarbose (5230 µg/mL), a antidiabetic drug widely used to treat postprandial hyperglycemia [128]. These findings can be compared with the values reported in other species of the Malvaceae family. For example, previous studies of *Ceiba pentandra* showed higher IC_50_ values than those reported in this study with methanolic extracts (86.49 µg/mL) and aqueous extracts (76.61 µg/mL) [12], as well as ethanolic fractions of *Sida linifolia* with IC_50_ value of 540 µg/mL (0.54 mg/mL) [54], while crude ethanolic extracts of *Hibiscus sabdariffa* reached values above 1000 µg/mL (1 mg/mL) [129]. On the other hand, Mayo-Montor et al. [130] also presented high IC_50_ values with methanolic extracts of *Sida rhombifolia* (330 µg/mL) and *Sida glabra* (>4000 µg/mL). In another study, Banerjee et al. [131] documented inhibition of this enzyme by ethanolic (IC_50_ = 74.15 µg/mL) and aqueous (IC_50_ > 200 µg/mL) extracts of *Abutilon indicum*. In comparison to the above, extracts from the leaves of *H. rovirosae* Standl showed better antihyperglucemic activity, as they managed to inhibit 50% of the α-glucosidase enzyme at considerably lower concentrations.

The inhibitory activity observed in *H. rovirosae* can be explained, at least in part, by its phytochemical profile dominated by glycosylated flavonoids, flavan-3-ols, and procyanidins, which were identified and quantified at high concentrations. In this regard, it is worth noting that the high abundance of quercetin-3-glucoside (155,930.2 ng/g) is particularly noteworthy, along with catechin (98,408.8 ng/g) and procyanidin B2 (85,661.7 ng/g), compounds widely recognized for their ability to inhibit enzymes involved in digestives [29,132]. Several reports in the literature also suggest that flavonoids such as quercetin (942.2 ng/g), kaempferol (408.9 ng/g), and luteolin (7145.8 ng/g), present in both *H. rovirosae* and other species of the Malvaceae family, such as *Grewia optiva* and *Ceiba pentandra*, can act as significant inhibitors of α-glucosidase [133,134].

In this context, previous findings have demonstrated that some phenolic derivatives can bind not only to the enzyme active site but also to allosteric regions of this, resulting in non-competitive inhibition and a subsequent reduction in carbohydrate digestion [135,136]. Similarly, some compounds of flavonol structures such as quercetin and Kaempferol act as non-competitive inhibitors by binding to various allosteric sites via hydrogen bonds [137,138]. Finally, molecular docking studies have revealed that some other flavonoids can also act as competitive inhibitors by mimicking the substrate and binding to the active site [139].

To date, no in vitro trials have been reported on the inhibition of these digestive enzymes by *H. rovirosae* Standl. Therefore, the results obtained in this study constitute an important precedent for this species, opening up the possibility of considering it as a potential natural source of therapeutic agents for managing postprandial hyperglycemia.

## 4. Conclusions

This research demonstrated that aqueous, ethanolic, and hydroethanolic extracts from *Hampea rovirosae Standl*. leaves are rich in phenols, flavonoids, and tannins, with the hydroethanolic extract containing the highest concentrations. The specific phytochemical profile revealed the predominance of quercetin derivatives, flavan-3-ols, procyanidins, and hydroxycinnamic and hydroxybenzoic acid. These metabolites are widely recognized for their antioxidant and antibacterial properties, as well as their strong potential for inhibiting digestive enzymes. All extracts exhibited strong antioxidant activity in the DPPH, ABTS, and FRAP assays, as well as marked inhibition of the α-glucosidase enzyme and moderate inhibition of α-amylase. Regarding the antibacterial activity, only the aqueous and hydroethanolic extracts exhibited inhibitory effects on the evaluated strains, with the aqueous extract being particularly relevant given its traditional use as an infusion. These findings provide valuable insight into the phytochemical and bioactive knowledge of *H. rovirosae*. However, further studies are required to isolate and identify the metabolites responsible for the effectiveness of this plant species and to confirm its effectiveness through in vivo assays. Overall, our results suggest a promising potential in the treatment of diabetes and antibacterial infections, as well as in the prevention and management of diseases associated with oxidative stress.

## Figures and Tables

**Table 1:** Quantitative profiling of phenolic acids and flavonoids compounds (ng/g) in the *H. rovirosae* leaf extracts.

Metabolite	Concentration in *H. rovirosae* Leaves Extracts (ng/g)
Quercetin-3-glucoside	155,930.2
Caffeic acid	134,399.1
Catechin	98,408.8
Procyanidin B2	85,661.7
Protocatechuic acid	83,824.3
Epicatechin	53,704.1
Ferulic acid	12,381.9
Gallic acid	12,216.6
Chlorogenic acid	8486.1
Luteolin	7145.8
p-Coumaric acid	4344.9
Proanthocyanidin A	4074.1
Apigenin	3588.9
Syringic acid	2116.1
Vanillic acid	1879.7
Naringenin chalcone	1401.5
Cinnamic acid	1102.7
Quercetin-3-galactoside	944.1
Quercetin	942.4
Kaempferol	408.9
Genistein	10.2
Daidzein	3.51
Naringenin	ND
Phloretin	ND
Resveratrol	ND
Rutin	ND

ND: Not detected.

**Table 2:** Extraction yield, total phenolic, flavonoid, and tannin content of *H. rovirosae Standl*. Leaves using different extraction solvents.

Extract	Yield %	Total Phenol Content (mg GAE/g Dry Extract)	Total Flavonoid Content (mg CE/g Dry Extract)	Total Tannin Content (mg GAE/g Dry Extract)
AE	33.50 ± 0.30 ^a^	252.15 ± 3.37 ^b^	46.76 ± 1.32 ^c^	196.39 ± 3.17 ^b^
EE	17.93 ± 0.40 ^b^	191.24 ± 3.71 ^c^	53.53 ± 1.32 ^b^	142.83 ± 2.34 ^c^
HEE	32.13 ± 0.20 ^a^	426.70 ± 1.06 ^a^	119.17 ± 0.90 ^a^	324.46 ± 5.31 ^a^

AE: aqueous extract; EE: ethanolic extract; HEE: hydroethanolic extract; GAE: gallic acid equivalent; CE: catechin equivalent. Values are means ± standard deviations of triplicates. Means in the same column followed by a different lowercase superscript letter are significantly different (*p* < 0.05).

**Table 3:** Antioxidant activity of *H. rovirosae Standl* extracts derived from leaves.

Extract	DPPH• (mg/mL, IC_50_)	ABTS•+ (mg/mL, IC_50_)	FRAP (µmol TE/g Ext)
AE	0.196 ± 0.00 ^b^	0.647 ± 0.01 ^c^	1032.66 ± 8.08 ^b^
EE	0.252 ± 0.01 ^c^	0.295 ± 0.00 ^a^	1045.33 ± 12.22 ^b^
HEE	0.110 ± 0.00 ^a^	0.507 ± 0.00 ^b^	2946.66 ± 30.55 ^a^

EE: stands for ethanolic extract; HEE for hydroethanolic extract, and AE for aqueous extract. The values are triplicate means ± standard deviations. A distinct lowercase superscript letter indicates a significant difference (*p* < 0.05) between means in the same column.

**Table 4:** Pearson correlation between the antioxidant activity and total TPC of *H. rovirosae Standl*. extracts.

Assay (Expressed in Units)	* TPC vs. Antioxidant Activity		
	Equation	Pearson’s Coefficient (r^2^)	Significance
DPPH (IC_50_)	y = 605.945 − 1640.72x	−0.988799	*p* < 0.001 (*p* = 0.0002)
ABTS (IC_50_)	y = 1320.61 − 1779.99x	−0.968378	*p* < 0.001 (*p* = 0.0015)
FRAP (µmol TE/g ext)	y = 62.0304 − 0.128841x	0.986957	*p* < 0.001 (*p* = 0.0003)

* Results in mg of GAE/g dry extract.

**Table 5:** Antibacterial activity of *H. rovirosae Standl.* extracts.

Extracts	Gram-Positive						Gram-Negative			
	*S. aureus*		*B. cereus*		*B. subtilis*		*S. typhimurium*		*E. coli*	
	DD	MIC/MBC	DD	MIC/MBC	DD	MIC/MBC	DD	MIC/MBC	DD	MIC/MBC
AE	-	-	-	-	-	-	9.1 ± 0.28	200/200	9.15 ± 0.49	200/200
EE	-	-	-	-	-	-	-	-	-	-
HEE	9.95 ± 0.0	60/60	12.15 ± 1.20	200/200	14 ± 1.27	400/400	-	-	-	-

EE: stands for ethanolic extract; HEE for hydroethanolic extract, and AE for aqueous extract; DD: diffusion on disc, MIC = minimum inhibitory concentration (mg/mL), MBC: minimum bactericidal concentration, inhibition zone expressed in mm. Values are means ± standard deviation of triplicates.

**Table 6:** Enzymatic assay using *H. rovirosae* Standl extracts.

Extracts	α-Amylase		α-Glucosidase	
	% Inhibition (1 mg/mL)	IC_50_ (mg/mL)	% Inhibition (50 µg/mL)	IC_50_ (µg/mL)
AE	14.38 ± 1.68 ^b^	3.08 ± 0.04 ^b^	36.44 ± 0.53 ^c^	66.01 ± 3.43 ^c^
EE	9.95 ± 1.73 ^c^	4.65 ± 0.05 ^c^	90.12 ± 2.25 ^b^	25.76 ± 2.06 ^b^
HEE	15.83 ± 1.07 ^b^	3.06 ± 0.01 ^b^	94.73 ± 1.42 ^a^	15.74 ± 0.43 ^a^
Acarbose	78.00 ± 2.89 ^a^	0.37 ± 0.02 ^a^	0.36 ± 0.00 ^d^	5230.00 ± 79 ^d^

EE: stands for ethanolic extract; HEE for hydroethanolic extract, and AE for aqueous extract. The values are triplicate means ± standard deviations. A significant difference (*p* < 0.05) between means in the same column is indicated by unique lowercase superscript letter.

## Data Availability

The original contributions presented in this study are included in the article/Supplementary Material. Further inquiries can be directed to the corresponding authors.

## Supplementary Materials

The following supporting information can be downloaded at: https://www.mdpi.com/article/10.3390/cimb48030327/s1. Supplementary Table S1. LOD (limits of detection), ULOQ, and LLOQ (upper and lower limits of quantification) of the target compounds. Supplementary Figure S1. Total Ion Chromatogram of *H. rovirosae*.
