# Supplementary material for: Phytochemical Profile and Bioactive Potential of Hampea rovirosae Standl.: Antioxidant, Antimicrobial, and Carbohydrate-Hydrolyzing Enzyme Inhibitory Activities
Source: Curr Issues Mol Biol. 2026 Mar 19;48(3):327. doi: 10.3390/cimb48030327 (PMC13025906; doi:10.3390/cimb48030327)
Supplement: Supplementary file 1 [file cimb-48-00327-s001.zip › cimb-4173587-supplementary.pdf]

Supplementary Table S1. LOD (limits of detection), ULOQ, and LLOQ (upper and lower limits of quantification) of the target compounds.

**Analyte**	**MW (g/mol)**	**LOD Conc in µM**	**ULOQ Conc µM**	**LLOQ Conc µM**	**CV (%)**
Apigenin	270.05	0.0097	5.23	0.0097	1.5
Caffeic acid	180.16	0.0019	1.05	0.0019	1.2
Catechin	290.26	0.0155	8.38	0.0155	5.3
Chlorogenic acid	354.31	0.0155	8.38	0.0155	5.7
Cinnamic acid	148.16	0.0116	6.29	0.0582	5.3
p-Coumaric acid	164.05	0.0039	2.10	0.0039	2.4
Daidzein	254.23	0.0116	6.29	0.0116	0.2
Epicatechin	290.26	0.0155	8.38	0.0776	0.4
Ferulic acid	194.18	0.0097	5.24	0.0097	1.7
Gallic acid	170.12	0.0116	6.29	0.0116	5.7
Genistein	270.24	0.0116	6.29	0.0116	9.0
Kaempferol	286.23	0.0116	6.29	0.0582	12.6
Luteolin	286.24	0.0097	5.24	0.0097	11.8
Naringenin	272.26	0.0039	2.10	0.0039	2.9
Naringenin chalcone	272.26	0.0097	5.24	0.0097	7.1
Proanthocyanidin A2	576.5	0.0155	8.38	0.0155	3.8
Procyanidin B2	578.5	0.0155	8.38	0.0155	3.4
Phloretin	274.26	0.0019	1.05	0.0019	2.8
Protocatechuic acid	154.12	0.0155	8.38	0.0155	4.2
Quercetin-3-glucoside	464.4	0.0155	8.38	0.0155	5.4
Quercetin-3-galactoside	464.4	0.0155	8.38	0.0155	0.4
Quercetin	302.24	0.0097	5.24	0.0097	10.3
Resveratrol	228.25	0.0097	5.24	0.0097	10.7
Rutin	610.52	0.0155	8.38	0.0155	3.2
Syringic acid	198.17	0.0116	6.29	0.0116	3.4
Vanillic acid	168.14	0.0155	8.38	0.0155	7.2

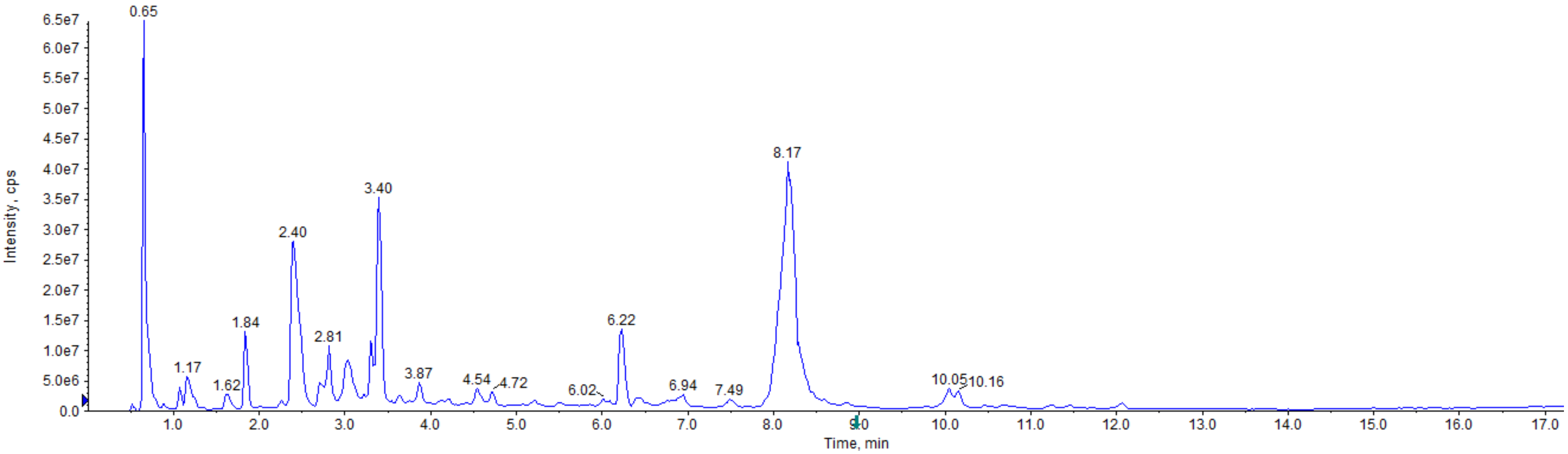

Supplementary Figure S1. Total Ion Chromatogram of *H. rovirosae*.
